# The Effect of Stroke Subtypes on Baroreceptor Sensitivity, a Predict for Acute Stroke Outcome

**DOI:** 10.1155/2019/7614828

**Published:** 2019-04-18

**Authors:** Wan-Chen Tsai, Hui-Chen Lin, Yun-Ru Lai, Che-Wei Hsu, Chih-Cheng Huang, Hung-Chen Wang, Chih-Min Su, Yu-Jih Su, Wei-Che Lin, Ben-Chung Cheng, Wen-Neng Chang, Cheng-Hsien Lu, Nai-Wen Tsai

**Affiliations:** ^1^Department of Neurology, Chang Gung Memorial Hospital-Kaohsiung Medical Center, Chang Gung University College of Medicine, Kaohsiung, Taiwan; ^2^Department of Biological Science, National Sun Yat-Sen University, Kaohsiung, Taiwan; ^3^Department of Neurosurgery, Chang Gung Memorial Hospital-Kaohsiung Medical Center, Chang Gung University College of Medicine, Kaohsiung, Taiwan; ^4^Department of Emergency Medicine, Chang Gung Memorial Hospital-Kaohsiung Medical Center, Chang Gung University College of Medicine, Kaohsiung, Taiwan; ^5^Department of Medicine, Chang Gung Memorial Hospital-Kaohsiung Medical Center, Chang Gung University College of Medicine, Kaohsiung, Taiwan; ^6^Department of Radiology, Chang Gung Memorial Hospital-Kaohsiung Medical Center, Chang Gung University College of Medicine, Kaohsiung, Taiwan

## Abstract

**Background:**

Reduced baroreflex sensitivity (BRS) has been reported in patients with acute cardiovascular events. We tested the hypothesis that BRS varies in different subtypes of acute ischemic stroke (AIS) and that BRS is a predictor of clinical outcomes.

**Methods:**

We examined autonomic parameters in 34 patients with AIS, including the small deep hemisphere infarction, the large hemisphere infarction, and the brainstem infarction groups on Day 1, Day 7, and Day 30 after AIS. Autonomic parameters were also evaluated in 18 age- and sex-matched healthy volunteers as a control group. The clinical outcomes were analyzed using the modified Rankin scale at 30 days after stroke.

**Results:**

The BRS, Valsalva ratio, and heart rate response to deep breathing (HR-DB) were significantly lower in patients after AIS on admission than in controls (*p*<0.01). The frequency domain of HRV (LF/HF ratio) was significantly increased in patients after AIS compared to controls (*p*<0.05). BRS was significantly reduced in patients with large hemisphere infarction or brainstem infarction compared to patients with small deep hemisphere infarction on Day 1 after AIS (*p*<0.01). Stepwise logistic regression showed that the levels of BRS and NIHSS are prognostic factors of 1-month outcomes in patients with AIS.

**Conclusion:**

Beside NIHSS score on admission, BRS is a potential prognostic factor of 1-month outcomes in patients with AIS. Patients with large hemisphere infarction or brainstem infarction have more blunting BRS than do those with lacunar infarction, which provides some insight into which patients may be expected to have a poor outcome.

## 1. Introduction 

Autonomic dysfunction is a common complication of acute ischemic stroke (AIS) [1, 2]. Previous studies have shown that autonomic dysfunction, including increased sympathetic activity and reduced baroreflex, may increase susceptibility to sudden death and predict adverse clinical outcomes after AIS [3, 4]. The possible mechanisms associated with autonomic impairment and poor outcome in AIS include increased cardiovascular events and progression of secondary brain injury due to inflammation and altered cerebral perfusion [4, 5].

The central autonomic network (CAN) includes central areas that interconnect with the autonomic nervous system (ANS) to regulate autonomic function. The areas of the CAN include the insular cortex, anterior cingulate cortex, amygdala, hypothalamus, periaqueductal gray, parabrachial nucleus, nucleus of the solitary tract, ventrolateral reticular formation of the medulla, and medullary raphe [6]. These areas generate stimulus-specific patterns of autonomic and neuroendocrine responses [7]. Recent studies have reported evidence that supratentorial infarction, especially in the right hemisphere, shows a tendency toward increased sympathetic activity [8, 9]. Brainstem stroke correlates with a significant reduction in parasympathetic and an increase in sympathetic influence that causes cardiovascular autonomic dysregulation [10, 11]. Accordingly, we speculated that the location of infarction is an important factor affecting autonomic cardiac dysfunction and impairment of the CAN in patients with AIS [12].

In this study, we hypothesized that different stroke localizations influence the development of different autonomic responses, measured by baroreflex sensitivity (BRS) and other cardiovascular autonomic function. The aim of this study was to determine whether BRS is distinct in stroke patients with different infarction locations and whether the BRS is a predictor of clinical outcomes.

## 2. Materials and Methods 

### 2.1. Study Participants

This prospective study that assessed the time course of cardiovascular function included 34 patients with AIS and 18 healthy volunteers at the Kaohsiung Chang Gung Memorial Hospital. The stroke diagnosis was based on clinical presentation, neurological examination, and results of brain magnetic resonance imaging (MRI) with diffusion-weighted images. Patients aged 18-85 years were classified into (1) large hemispheric infraction, (2) small deep hemispheric infarction, and (3) brainstem infarction group in the study [13]. The hospital's Institutional Review Committee on Human Research approved the study protocol, and all participants provided informed consent.

Patients with intracranial hemorrhage were excluded, as well as those with underlying neoplasm, vasculitis, hematological disorders, end-stage renal disease, liver cirrhosis, or congestive heart failure. Patients with cardioembolic stroke, as well as those who received intravenous thrombolytic therapy, were excluded because those patients have different therapeutic strategies and a high percentage of hemorrhagic transformation. Cardioembolism was diagnosed by clinical presentation, electrocardiography (ECG), and cardiac ultrasound.

### 2.2. Clinical Assessments

The clinical assessments followed our previous method [14]. All patients underwent complete neurological examination and detailed medical history upon enrollment and during follow-up. Brain MRI with MR angiography, extracranial carotid sonography, and transcranial color-coded sonography was performed on patients with AIS. Demographic data, history of previous vascular events (i.e., myocardial infarction, coronary artery disease, and previous stroke), and vascular risks (i.e., hypertension, diabetes mellitus (DM), dyslipidemia, and smoking) were obtained at baseline [15].

Physical disability and handicap were evaluated using the modified Rankin scale (mRS). The mRS was evaluated by investigators (Dr. Yu-Jun Lin and Yun-Ru Lai) blinded to the study group of each subject. Functional outcomes were evaluated at one month after stroke. A good outcome was defined as mRS score of 0-3 without any cardiovascular event, while a poor outcome was defined as mRS score of 4-6 [16].

### 2.3. Assessment of Autonomic Function

The methodology of autonomic functional assessment was modified from our previous publication [17]. All autonomic function studies were conducted on Days 1, 7, and 30 after stroke. The schedule was arranged between 8 and 10 AM to avoid the circadian effect. Coffee, food, alcohol, and nicotine were not permitted for 4 hours before the examination. Patients on medications known to cause orthostatic hypotension or otherwise affect autonomic testing were asked to abstain. Medication for BP control was continued, except for *β*-blockers, which were ceased on the day of the study and resumed after the test.

Heart rate was continuously recorded from standard three-lead ECG (Ivy Biomedical, Model 3000; Branford, CT, USA), and arterial BP was continuously measured using beat-to-beat photoplethysmographic recordings (Finameter Pro, Ohmeda; Englewood, OH, USA). The following parameters were obtained through tests computed by Testworks (WR Medical Electronics Company, Stillwater, MN, USA): HR response to deep breathing (HR_DB) and Valsalva ratio (VR) [18].

Beat-to-beat R-R interval (RRI) changes were interpolated using a third-order polynomial and were resampled with an interval of 0.5s. For spectral analysis of heart rate variability (HRV), the signals were then transformed to the frequency domain with fast Fourier transformation using 512 samples. Spectral powers were divided into three frequency domains: high frequency (HF, 0.15–0.4 Hz), low frequency (LF, 0.04–0.15 Hz), and very low frequency (VLF, 0–0.04 Hz). The unit of RRI power is ms^2^. The normalized low and high frequency powers (LF normalized units, HF normalized units) were calculated as a percentage of overall power.

Spontaneous BRS was computed by using Nevrokard sequence BRS program [19]. The criteria in computing BRS, (1) systolic BP (SBP) changes > 1 mmHg, (2) sequences longer than 3 beats, and (3) correlation coefficient > 0.85. Both bradycardic (an increase in SBP that caused an increase in RRI) and tachycardic sequences (a decrease in SBP that caused a decrease in RRI) that fulfilled the criteria, were enrolled. The BRS was calculated using a synchronous mode and a shift mode from 1 to 6 heart beat shifts [20, 21] for each subject. The mode with the largest number of slopes was selected and the average slope of the regression lines was taken as the measure of BRS.

### 2.4. Statistical Analyses

The quantitative data are presented as mean ± standard deviations (SD). Continuous variables between two groups (i.e., controls vs. patients, good and poor outcome group) were compared using the independent* t*-test for parametric data and the Mann-Whitney U test for nonparametric data. Chi-square or Fisher's exact test was used for the comparison of proportions between two groups. One-way ANOVA was used to compare the autonomic parameters among the three stroke subtypes. Repeated measures ANOVA was used to compare BRS and HR-DB at different time points (on Days 1, 7, and 30 post-stroke), while Scheffe's multiple comparison was performed to analyze the intraindividual course of parameters over time and compare the parameters of the good and poor outcome groups. The independent* t*-test was also used to compare the good and poor outcome groups. Multiple logistic regression analyses were used to determine the independent influence of different predictive variables on functional outcome. Statistical significance was set at* p*<0.05. All statistical calculations were performed using the SAS software package, version 9.1 (2002, SAS Statistical Institute, Cary, NC, USA).

## 3. Results

### 3.1. Demographic Data of Stroke Patients and Controls

The demographic data of the AIS patients and controls are shown in Table 1. The 34 patients with AIS (mean age: 63.2 years) included 26 males and 12 females. Age and sex were similar between the two groups. Twenty-eight subjects had one or more underlying diseases, including hypertension (26), diabetes mellitus (19), hyperlipidemia (23), coronary artery disease (2), and smoking (6). The blood pressure, serum triglycerol, HbA1c, and high-sensitivity C-reactive protein (hs-CRP) levels were significantly higher in the AIS patient group than in the control group (*p*<0.05).

### 3.2. Comparison of Autonomic Function between Stroke Patients and Controls

In terms of autonomic function testing, VR, HR_DB, and spontaneous BRS were significantly reduced in the stroke group compared to the control group (*p*<0.01). The normalized frequency power (LF/HF ratio) was significantly higher in the AIS patient group than in the control group (*p*<0.05). There were no significant differences in white blood cell counts (WBC), red blood cell counts (RBC), platelet counts, or serum total cholesterol and LDL-cholesterol levels.

### 3.3. Comparison of Autonomic Function among 3 Subtypes of Acute Ischemic Stroke

**Figure 1:**
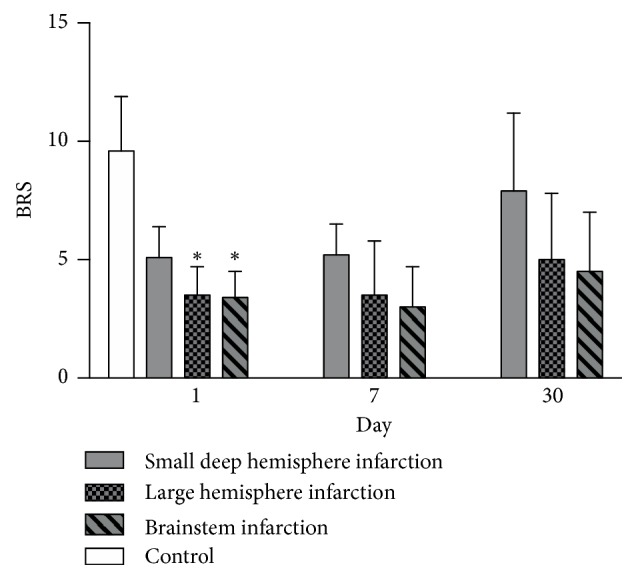
Changes in BRS level among control and the 3 stroke subtypes (small deep hemisphere infarction, large hemisphere infarction and brainstem infarction) on Day 1, Day 7, and Day 30 after acute ischemic stroke. *∗p*<0.05 compared to the small deep hemisphere infarction.

The basic demographic, stroke locations and autonomic function among 3 subtypes of stroke patients are listed in Table 2. The subtypes of AIS were small deep hemisphere infarction (14 patients), large hemisphere infarction (13 patients), and brainstem infarction (7 patients). The mall deep hemisphere infarction group had significantly higher BRS on Day 1 after stroke compared to both the large hemisphere infarction group and the brainstem infarction group. There was no statistical difference between the left and the right infarcts in each group. We had 4 patients (2 in the right hemisphere and 2 in the left hemisphere) involving insular infarction, but there was no statistical difference in autonomic parameters between the left and the right sides. Furthermore, the differences in BRS between the three subtypes were not significant on Day 7 or Day 30 after stroke (Figure 1). There were also no significant differences in the spectral analysis (LF, HF, and LF/HF ratio) parameters between the three groups.

### 3.4. Prognostic Factors of Clinical Outcome

The potential prognostic factors of the 34 acute stroke patients for one month are listed in Table 3. Among the patients, 24 had good outcomes and the remaining 10 had poor outcomes, but no patients died during the follow-up period. Statistical analysis revealed that NIHSS, BRS, and HR_DB were potential predictors of outcomes. However, age, sex, underlying disease, and laboratory data were not significantly different between the good and the poor outcome groups. Using the stepwise logistic regression model for these variables, only BRS (OR= 4.1, 95% CI 1.3-13.1) and NIHSS on admission (OR= 0.71, 95% CI 0.53-0.95) were independently associated with one-month outcome.

### 3.5. Time Course of BRS Change

**Figure 2:**
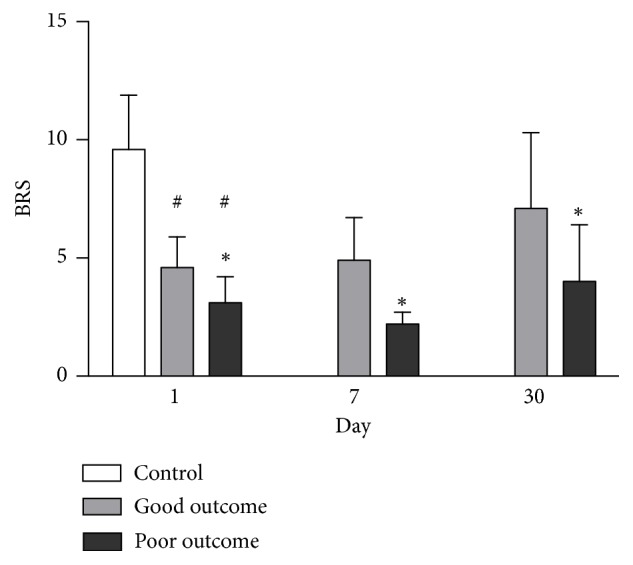
Comparison of BRS level between the good outcome group and the poor outcome group on Day 1, Day 7, and Day 30 after acute ischemic stroke. *∗p*<0.05 compared to the good outcome group. ^**#**^*p*<0.05 compared to the control group.

**Figure 3:**
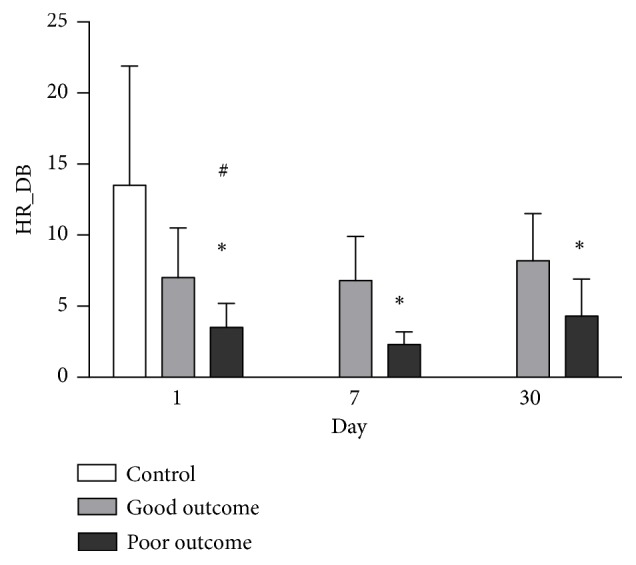
Comparison of HR_DB level between the good outcome group and the poor outcome group on Day 1, Day 7, and Day 30 after acute ischemic stroke. *∗p*<0.05 compared to the good outcome group. ^**#**^*p*<0.05 compared to the control group.

The BRS values in AIS patients were initially lower and gradually increased after acute stroke compared to the control group. Furthermore, BRS values were significantly lower in patients with poor outcomes than in those with good outcomes on Day 1, Day 7 and even Day 30 (Figure 2). Similarly, the HR_DB values in patients with poor outcomes were significantly lower than those in patients with good outcomes on Day 1, Day 7 and Day 30 (Figure 3).

## 4. Discussion

The present study examined autonomic function and different subtypes of AIS and has four major findings. First, the baroreflex function (spontaneous BRS) and cardiovagal autonomic function (Valsalva ration and HR-DB) were significantly lower in patients with AIS on admission than in controls. Second, the frequency domain of HRV (LF/HF ratio) is significantly increased in patients with AIS compared to controls. Third, the values of BRS are significantly reduced in both large hemisphere infarction and brainstem infarction compared to small deep hemisphere infarction after AIS. Finally, the levels of BRS and NIHSS are potential prognostic factors of 1-month outcomes in patients with AIS.

BRS provides regulation of cardiovagal control and the interaction of cardiac sympathetic–parasympathetic balance. Impairment of BRS tended to have sympathetic hyperactivity, which consequently leads to an increased risk of cardiac arrhythmia, hypertensive crisis, and associated cardiac events. Furthermore, BRS impairment results in BP instability, which is likely to have a negative impact on cerebral blood perfusion, especially in patients with impaired cerebral autoregulation, such as AIS patients [22]. These mechanisms may explain the poor prognosis of the patients with impaired BRS.

A previous study has demonstrated that reduced BRS in the acute phase of stroke can predict long-term fatalities. Another study has shown that autonomic dysregulation can persist 9 months after stroke [23]. Our previous study confirmed the hypothesis that autonomic dysregulation can persist for a long time among AIS survivors and demonstrates that patients with recurrent cardiovascular events after symptomatic AIS have significantly lower HR_DB, with a trend toward reduced VR and BRS [21]. In addition to the findings in patients with AIS, our recent research shows the predictive value of BRS in patients with spontaneous intracranial hemorrhage [24]. In the current study, the BRS values in the poor outcome group were significantly lower than those in the good outcome group on Day 1, Day 7, and Day 30. Our results are in agreement with those of previous studies that show BRS correlated to clinical outcome in patients with AIS [22, 25]. Based on this finding, it is recommended to test patients within 24 hours of symptom onset in further studies of BRS or other cardiovascular autonomic changes in stroke patients to observe the most prominent differences as these differences may diminish gradually.

The CAN, which includes the insular cortex, anterior cingulate cortex, amygdala, hypothalamus, periaqueductal gray, parabrachial nucleus, nucleus of the solitary tract, ventrolateral reticular formation of the medulla, and medullary raphe, is a group of central areas that interconnect with the peripheral ANS to regulate autonomic function [6]. Baroreceptor signaling is conducted via vagal and glossopharyngeal cranial nerves to the nucleus tractus solitarius, which in turn projects to the brainstem (e.g., ventrolateral medulla and nucleus ambiguus). The brainstem is a critical part of the baroreflex arc and participates in homeostatic tuning of the BRS. Accordingly, we speculated that the location of infarction is an important factor affecting autonomic dysfunction and CAN impairment in patients with AIS [12]. Recent evidence reported that supratentorial infarction, especially in the right hemisphere, shows a tendency toward increased sympathetic activity [8, 9]. However, our study does not show the difference of autonomic parameter between the left and the right infarcts in each group. We propose that our case number is too small to clarify this issue. Brainstem stroke correlates with a reduction in parasympathetic and an increase in sympathetic influence that caused cardiovascular autonomic dysregulation [10, 11].

The degree of autonomic dysregulation can also be influenced by stroke subtypes. One study revealed that the autonomic dysfunctions are influenced by stroke subtype, with large hemisphere arthrosclerosis showing more severe impairment of parasympathetic function [26]. Other studies have demonstrated that insults to both the brainstem and the hemispheres can result in HRV impairment [27, 28], not only in the acute phase but also on long-term follow-up. Impaired HRV is associated with severe neurological deficits and disability [29]. Low HR_DB and VR and a trend toward reduced HF in the HRV frequency domain of AIS patients are consistent with impaired cardiovagal function. This finding is corroborated by the present study that shows that patients with either large hemisphere infarction or brainstem stroke had significant reduction of BRS compared to those with small deep hemisphere infarction after AIS.

This study has several limitations. First, patients who were comatose and those who were unable to cooperate for the autonomic function test were excluded. Thus, the role of the BRS in critically ill and high-risk patients is unknown. Second, we did not enroll patients with cardioembolic stroke in this study because both BRS and other cardiovagal autonomic parameters may be influenced by the effects of cardiac arrhythmia, which would result in unreliable data. Third, some autonomic parameters may be influenced by the effects of medications (e.g., calcium channel blockers; angiotensin II receptor antagonist, *β*-blockers), which are commonly used by AIS patients and were not stopped during the study for ethical reasons. Furthermore, the area of each patient's cerebral infarction is not the same and the number of cases in the single stroke location is small; it is difficult to analyze the correlation between single infarct location and autonomic parameters. Finally, the number of variables considered for multiple logistic regression analysis is small and only one variable was selected as important in predicting outcome in the stepwise procedures. Thus, the maximum likelihood estimates of the coefficients are only valid in the current analysis.

In conclusion, the measurement of BRS is a potential prognostic factor of short-term outcome in patients with AIS. Patients with large hemisphere infarction or brainstem infarction have more blunting BRS than those with lacunar infarction, which may suggest that those patients are expected to have a poor outcome. The successful translation of these results may offer useful predictors of short-term outcomes after AIS.

## Figures and Tables

**Table 1:** Baseline characteristics and autonomic function of the study participants.

	Stroke patients (n=34)	Controls (n=18)	*p*-value
Age (y) (mean ±SD)	63.2±8.7	59.6±7.8	0.14
Male/female	26/12	11/7	0.138
Mean systolic blood pressure	169.4±28.4	126.70±13.1	<0.0001*∗*
Mean diastolic blood pressure	92.5±15.5	68.15±6.1	<0.0001*∗*
*Underlying diseases*			
Hypertension (n,%)	26 (76.5%)	---	
Diabetes mellitus (n,%)	19 (55.9%)	---	
Dyslipidemia (n,%)	23 (67.6%)	---	
Coronary artery diseases (n, %)	2 (5.9%)	---	
Smoking	6 (17.8%)	---	
*Laboratory data*			
White blood cells(×10^3^/ml)	9.4±1.7	5.3±1.2	0.08
Red blood cells (×10^6^/ml)	4.7±0.6	4.7±0.7	0.68
Platelet counts (×10^4^/ml)	20.4±6.6	24.4±7.5	0.06
Hs-CRP (mg/L)	6.1±2.6	0.8±0.2	0.008*∗*
Total cholesterol (mg/dL)	181.2±39.4	189.9±27.9	0.42
LDL-cholesterol (mg/dL)	106.9±31.9	111.9±25.8	0.58
Triglycerol (mg/dL)	165.2±128.9	92.8±48.4	0.03*∗*
HbA1c (%)	7.5±2.0	5.8±0.3	0.002*∗*
*Autonomic function*			
Spectral analysis			
LF (n.u.)	59.6±22.6	50.2±18.3	0.14
HF (n.u.)	40.2±22.4	49.7±18.3	0.13
LF/HF ratio	2.4±2.2	1.3±1.2	0.04*∗*
Cardio-vagal autonomic function			
Valsalva ratio	1.1±0.3	1.7±0.4	0.004*∗*
HR_DB	6.4±3.5	13.5±8.4	0.006*∗*
Baroreflex function			
BRS	4.2±1.4	9.6±2.3	<0.0001*∗*

All values are presented as mean ± SD; *∗p* < 0.05, compared to controls.

*Abbreviations.* HbA1c: glycated hemoglobin; Hs-CRP= high-sensitivity C-reactive protein; LDL: low density lipoprotein; SD: standard deviation; LF: low frequency; HF, high frequency; HR_DB: heart rate response to deep breathing; BRS: baroreflex sensitivity; n.u.: normalized unit.

**Table 2:** The basic demographic, stroke locations, and autonomic function among 3 subtypes of stroke patients.

	Small deep hemisphere infarction (n=14)			Large hemisphere infarction (n=13)			Brainstem infarction (n=7)			*p*-value
	Left	Right	Total	Left	Right	Total	Left	Right	Total	
*Age*			*61.7±9.9*			*63.5±8.7*			*65.6±5.8*	*0.63*
*Sex (female) n*			*6*			*4*			*2*	*0.34*
*NIHSS*			*4.3±1.5*			*5.3±5.3*			*10.4±8.3*	*0.04*
*SBP*			*172.4±24.7*			*163.2±29.6*			*175.0±34.8*	*0.60*
*DBP*			*95.8±15.9*			*89.5±17.5*			*91.4±11.0*	*0.58*
*Lateralization*	6	8		6	7		4	3		
*Stroke location*										
Internal capsule	2	2	*4*							
Basal ganglia	2	5	*7*							
Corona radiate	3	5	*8*							
Putamen	0	1	*1*							
Frontal lobe				5	3	*8*				
Temporal lobe				4	3	*7*				
Insula				2	2	*4*				
Parietal Lobe				2	5	*7*				
Occipital lobe				0	2	*2*				
Midbrain							1	0	*1*	
Pons							3	3	*6*	
Medulla							0	2	*2*	
*Spectral analysis*										
LF (n.u)	49.0±22.0	63.9±16.1	*57.5±19.6*	59.5±19.1	57.1±25.9	*58.2±22.1*	82.0±6.7	45.0±39.1	*66.1±30.5*	*0.69*
HF (n.u)	50.8±21.8	35.7±16.1	*42.2±19.6*	40.6±19.0	42.5±25.6	*41.6±21.9*	28.7±6.5	45.0±39	*33.8±30.1*	*0.71*
LF/HF ratio	1.2±1.1	2.6±2.1	*2.0±1.9*	2.2±2.0	2.3±2.2	*2.3±2.0*	4.2±2.5	2.7±1.9	*3.7±2.8*	*0.24*
*Cardio-vagal function*										
Valsalva ratio	1.3±0.6	1.0±0.3	*1.2±0.4*	1.0±0.1	1.0±0.3	*1.0±0.2*	1.1±0.4	1.1±0.2	*1.1±0.3*	*0.41*
HR_DB	6.8±1.3	9.0±4.7	*7.8±3.3*	5.3±3.4	5.6±3.4	*5.4±3.2*	3.0±0.6	2.0±0.8	*2.5±0.7*	*0.07*
*Baroreflex function*										
BRS	4.5±1.0	5.6±1.3	*5.1±1.3*	4.0±3.4	3.1±0.9	*3.5±1.2∗*	3.5±1.0	3.3±1.5	*3.4±1.1∗*	*0.002§*

All values are mean ± SD; **§ ***p*-value are compared with overall mean among 3 groups; *∗p* < 0.05, compared to small deep hemisphere infarction with post hoc analysis.

Because some patients have multiple infarction, the total number of the stroke locations is greater than the number of patients.

*Abbreviations.* SD: standard deviation; LF: low frequency; HF: high frequency; HR_DB: heart rate response to deep breathing; BRS: baroreflex sensitivity n.u.: normalized unit

**Table 3:** Prognostic factors of patients with acute ischemic stroke.

	Good outcome (n=24)	Poor outcome (n=10)	*p*-Value	Adjusted OR (95% CI)	Adjusted *p*-Value
*Age (y)*	62.0±9.0	66.1±7.2	0.21		
*Sex (Male/female)*	17/7	9/1	0.23		
*Underlying diseases*					
Hypertension	20	6	0.20		
Diabetes mellitus	12	7	0.45		
Coronary artery disease	1	1	0.51		
Hyperlipidemia	17	6	0.54		
Smoking	5	1	0.76		
*Clinical feature*					
Systolic blood pressure on admission	171.4±27.0	164.7±32.3	0.54		
Diastolic blood pressure on admission	94.6±15.1	87.4±16.2	0.22		
NIHSS	4.1±2.1	10.3±8.2	0.001	0.71(0.53-0.95)	*0.021*
*Laboratory data*					
White blood cells(×10^3^/ml)	14.0±1.7	13.5±1.3	0.87		
Red blood cells (×10^6^/ml)	4.7±0.6	4.6±0.7	0.77		
Platelet counts (×10^4^/ml)	21.1±7.1	18.7±5.3	0.34		
Hs-CRP (mg/L)	5.9±2.9	7.0±3.0	0.81		
Total cholesterol (mg/dL)	186.1±42.0	171.5±33.5	0.35		
LDL-cholesterol (mg/dL)	111.2±32.8	98.5±30.2	0.32		
Triglycerol (mg/dL)	165.4±30.8	165.0±36.6	0.99		
HbA1c (%)	7.4±2.1	7.9±1.9	0.74		
*Autonomic function on admission*					
Frequency domain					
LF (n.u)	58.3±22.8	62.6±23.1	0.62		
HF (n.u)	41.4±22.6	37.4±22.8	0.64		
LF/HF ratio	2.2±1.9	3.0±2.9	0.32		
Cardio-vagal autonomic function					
Valsalva ratio	1.1±0.3	1.3±0.4	0.52		
HR_DB	7.0±3.5	3.5±1.7	0.02		
Baroreflex function					
BRS	4.6±1.3	3.1±1.1	0.003	4.1(1.3-13.1)	*0.016*

All values are mean ± SD; adjusted *p*-value was calculated by using the stepwise logistic regression model for the potential variables.

*Abbreviations.* HbA1c: glycated hemoglobin; Hs-CRP= high-sensitivity C-reactive protein; LDL: low density lipoprotein; SD: standard deviation; LF: low frequency; HF: high frequency; HR_DB: heart rate response to deep breathing; BRS: baroreflex sensitivity; n.u.: normalized unit.

## Data Availability

The data used to support the findings of this study are available from the corresponding author upon request.

## Abbreviations

AIS: Acute ischemic stroke
ANS: Autonomic nervous system
BP: Blood pressure
BRS: Baroreflex sensitivity
CAN: Central autonomic network
DM: Diabetes mellitus
ECG: Electrocardiography
HbA1c: Glycolate hemoglobin
HF: High frequency
HR-DB: Heart rate response to deep breathing
HRV: Heart rate variability
hs-CRP: High sensitivity C-reactive protein
LDL: Low density lipoprotein
LF: Low frequency
mRS: Modified Rankin Scale
OR: Odds ratio
RBC: Red blood cell
RRI: R-R interval
SD: Standard deviation
VLF: Very low frequency
VR: Valsalva ratio
WBC: White blood cell.
